# Polyphenolic and molecular variation in *Thymus* species using HPLC and SRAP analyses

**DOI:** 10.1038/s41598-021-84449-6

**Published:** 2021-03-03

**Authors:** Danial Sarfaraz, Mehdi Rahimmalek, Ghodratollah Saeidi

**Affiliations:** 1 Department of Agronomy and Plant Breeding, College of Agriculture, Isfahan University of Technology, 84156 83111 Isfahan, Iran; 2 Department of Horticulture, College of Agriculture, Isfahan University of Technology, 84156-83111 Isfahan, Iran

**Keywords:** Genetic markers, Chemical ecology

## Abstract

In the present research, inter and intra genetic variability of 77 accessions belonging to 11 *Thymus* species were assessed using eight SRAP primer combinations. High polymorphism (98.3%) was observed in the studied species. The cluster analysis classified *Thymus* species into five main groups. According to molecular variance (AMOVA) analysis, 63.14% of total genetic variation was obtained within the species, while 36.86% of variation was observed among species. STRUCTURE analysis was also performed to estimate the admixture of species. For instance, *T. carmanicus* and *T. transcaspicus* revealed high admixtures. HPLC analysis also demonstrated the presence of rosmarinic acid (32.3–150.7 mg/100 g DW), salvianolic acid (8–90 mg/100 g DW), and cinnamic acid (1.7–32.3 mg/100 g DW) as major phenolic acids, as well as apigenin, epicatechin, and naringenin as the major flavonoids. The highest phenolic and flavonoid contents were detected in *T. transcaspicus* (37.62 mg gallic acid equivalents (GAE) g^−1^ DW) and *T. vulgaris* (8.72 mg quercetin equivalents (QE) g^−1^ DW), respectively. The antioxidant properties and total phenolic of *Thymus* species were examined using DPPH and β-carotene-linoleic acid model systems and consequently *T. vulgaris* and *T. pubescens* were detected with the highest and the lowest antioxidant activities respectively. Cluster and principal Components Analysis (PCA) of the components classified the species in to three groups. Finally, similarity within some species was observed comparing molecular and phytochemical markers. For instance, *T. vulgaris* separated from other species according to major polyphenolic profiles and molecular analyses, as well as *T. transcaspicus*, *T. carmanicus*, and *T. fedtschenkoi* that were clustered in the same groups.

## Introduction

Phenolic and flavonoid compounds have been known as natural products with high antioxidant activity. Nowadays, such components are frequently used and emphasized in food and some industrial products because of their health properties^1^. These compounds also have a crucial role in scavenging of the free radicals that are considered as serious risk factors for human health. Therefore, polyphenolic components extracted from natural sources of plant species are of great importance and thence, there is a growing interest to use the natural sources of antioxidants instead of synthetic ones.

Thyme (*Thymus* spp.) has been used as one of the most substantial medicinal plants for food and pharmaceutical purposes^2^. *Thymus* is considered as one of the most important genera of the Lamiaceae family with more than 100 species^3^. It is a perennial medicinal plant wildly grown in different regions of the world. Mediterranean regions have been assumed as the origin of this plant^4^. *Thymus* species has been applied in food industries for different purposes including flavoring agent, herbal tea and new products with low antibacterial activities. Furthermore, thyme has been used as antifungal, antiviral, anti-inflammatory, anti-parasitic, and spasmolytic^5^. Most of the studies on the *Thymus* species have focused on essential oil components of the species in different countries, including Iran^2,3^, Serbia^6^, and Ethiopia^7^. However, there are limited reports in respect to polyphenolic profiles of *Thymus* species, including *T. vulgaris*^8^, *T. satureioides*^9^, *T. pannonicus*^10^, and *T. praecox*^11^ using chromatography-based analysis. Phenolic acids as well as flavonoids and their derivatives display high variations in different *Thymus* species such as *T. algeriensis* (rosmarinic acid and kaempferol-O-glucuronide)^12^, *T. pulegioides* (rosmarinic acid and luteolin derivatives)^13^, and *T. capitatus* (rosmarinic acid, salvianolic acid)^14^. In Iran, 14 *Thymus* species have been identified out of which four of are known to be endemic^15^. However, there are no reports regarding phenolic and flavonoid components of Iranian *Thymus* species based on HPLC analysis. Furthermore, most of the previous researches have focused on polyphenolic profiles of one or few limited species and there are no comprehensive and comparative study focusing on phenolics, flavonoids and their derivatives in *Thymus* species.

Besides the polyphenolic profiles, molecular studies can provide new insights for further classification and breeding programs in this genus. SRAP (Sequence Related Amplified Polymorphism) is a PCR-based dominant marker that amplifies the fragments selectively by targeting the functional genome regions. This marker has been used to amplify the Open Reading Frame (ORF)^16^. This method is simple and the bands can be easily scored and interpreted^17^. Previous reports displayed higher polymorphism nature of SRAP markers in comparison with other dominant markers such as ISSR and RAPD^18,19^. Finally, SRAP has been considered as a high-throughput marker for studying genetic diversity, which has not yet been studied among and within *Thymus* species. SRAP marker has been applied for the assessment of the genetic diversity in many Lamiaceae plants including *Origanum* genus^20^, *Salvia aristata*^21^, and *Satureja* species^22^. There are limited reports on genetic diversity of *Thymus* species and most of researches focused on one species including *T. daenensis*^15,23,24^, *T. sibthorpii*^25^, *T. carmanicus*^26^, *T. kotschyanus* and *T. vulgaris*^27^. Moreover, there are limited reports in respect to HPLC analysis of *Thymus* species including *T. serpyllum* and *T. argaeus*^28,29^. In the present study, the natural compounds of several *Thymus* species were introduced for the first time. Moreover, as the previous studies mostly reported one species and the extraction methods were different, so another importance of this research was the comparison of 11 *Thymus* species in one experiment using a similar extraction method that increase the validity of comparison. Therefore, this is the first report to introduce and compare the phenolic and flavonoid compounds of several *Thymus* species based on HPLC analysis. Finally, comparing phytochemical and molecular markers can contribute to the improvement of the components as well as breeding of studied *Thymus* species.

The objectives of the present study are: (1) to compare total phenolic, flavonoid and antioxidant capacity of 11 *Thymus* species viz. *Thymus* *migricus* Klokov & Des. -Shost., *T. fallax* Fisch. & C. A. Mey., *T. serpyllum* L., *T. trautvetteri* Klokov & Desj. -Shost., *T. transcaspicus* Klokov, *T. carmanicus* Jalas, *T. fedtschenkoi* Ronneger, *T. daenensis* Celak *subsp. daenensis*, *T. pubescens* Boiss. & Kotschy ex Celak*, T. kotschyanus* Boiss. & Hohen., and *T. vulgaris* L, (2) to determine the polyphenolic profiles of studied species based on HPLC analysis, (3) to determine the inter- and intra-genic relationships of the species based on SRAP molecular marker, and (4) to classify the species based on molecular and phytochemical markers using multivariate analyses.

## Results and discussion

### Phytochemical analysis

**Figure 1:**
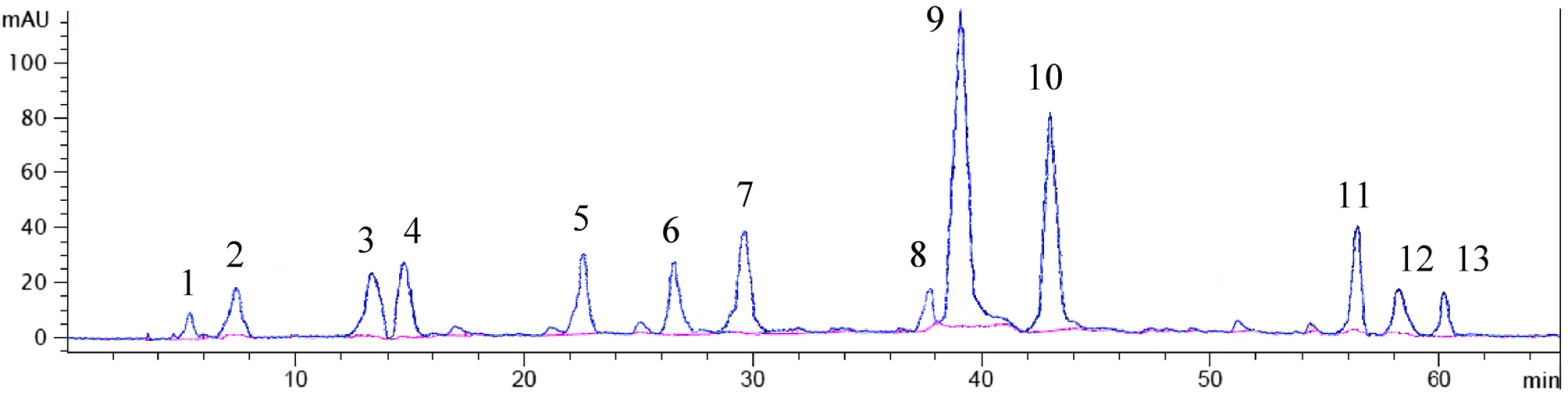
HPLC chromatogram of *Thymus trautvetteri*. The polyphenolic compounds were described in footnote. Footnote: 1: gallic acid, 2: epicatechin, 3: chlorogenic acid, 4: caffeic acid, 5: luteolin-7-o-glucoside, 6: p-coumaric acid, 7: ferulic acid, 8: cinnamic acid, 9: rosmarinic acid, 10: salvianolic acid, 11: apigenin, 12: naringenin, 13: kaempferol.

Quantitative determination of phenolic acids, flavonoid compounds, and their derivatives were performed using HPLC analysis (Table 1, Fig. 1). HPLC results showed high variation among studied species. Rosmarinic acid, salvianolic acid, cinnamic acid, ferulic acid, caffeic acid apigenin were the major polyphenolic components of *Thymus* methanolic extract in 11 assessed species. Among phenolic acids, rosmarinic acid possessed the highest content, while among flavonoids apigenin revealed the highest values. Rosmarinic acid has been reported as the major phenolic acid in most of the previous studies in several species^9,10,30^, while different classes of flavonoids have been reported in *Thymus* species. Rosmarinic acid content ranged from 32.2 mg/100 g DW in *T. daenensis* to 150.7 mg/100 g DW in *T. serpyllum* (Table 1). Similarly, Zengin et al.^29^ reported a higher content of rosmarinic acid in *T. argaeus* as compared to our studied species by using similar methanolic extraction method, column (C18) and standard compounds. However, the extraction method and the harvesting time are reported as two most determining factors that can highly affect the chromatography output^31^. Rosmarinic acid can be found in some other Lamiaceae plants such as *Origanum vulgare*, *Salvia officinalis*^28^, *Ocimum basilicum*^32^ and *Agastache rugosa*^33^. It has valuable properties such as anti-viral, anti-thrombotic, anti-inflammatory and antiglycative activities^34,35^. Salvianolic acid also showed high amounts compared to other phenolic acids. The highest and the lowest amounts of salvianolic acid were obtained in *T. vulgaris* (90.0 mg/100 g DW) and *T. migricus* (8.0 mg/100 g DW), respectively. This compound is also considered as one of the major phenolic acids in other reported *Thymus* species, including *T. capitatus*^14^, *T. carnosus*^36^ and *T. pannonicus*^10^. Various pharmaceutical properties have been reported for salvianolic acid including anti-inflammatory^37^, anti-diabetic activity^38^, as well as cardiovascular effects. This compound has also been found in other Lamiaceae species, as is considered in the main phenolic acid component of *Salvia miltiorrhiza*^38^.

**Table 1:** Major phenolic and flavonoid compounds of the studied *Thymus* species based on HPLC analysis.

Standards	RT^a^	*T. carmanicus*	*T. daenensis*	*T. fallax*	*T. fedtschenkoi*	*T. kotschyanus*	*T. migricus*	*T. pubesence*	*T. trautvetteri*	*T. serpyllum*	*T. transcaspicus*	*T. vulgaris*
Gallic acid	5.5	3.0	3.6	nd^b^	2.8	0.0	2.4	0.8	0.3	2.0	1.3	5.5
Epicatechin	7.41	1.7	1.7	1.5	1.9	1.7	1.1	1.1	0.9	0.5	1.7	2.3
Chlorogenic acid	13.35	6.0	3.3	nd^b^	nd^b^	nd^b^	nd^b^	nd^b^	2.9	nd^b^	4.2	nd^b^
Caffeic acid	14.68	27.4	13.1	12.9	14.0	14.2	14.0	15.8	15.8	16.0	13.7	11.8
Luteolin-7-o-glucoside	22.56	7.9	3.3	1.9	43.9	12.7	4.5	6.1	11.0	2.4	1.7	0.8
p-Coumaric acid	26.5	3.6	5.7	7.5	4.4	8.8	6.1	9.0	6.9	6.3	nd^b^	2.9
Ferulic acid	29.74	14.2	16.3	15.1	10.3	19.6	12.9	16.7	15.2	13.2	12.2	25.6
Cinnamic acid	37.96	1.7	30.7	10.7	12.1	7.6	6.8	5.4	4.3	7.9	1.7	32.3
Rosmarinic acid	39.04	88.0	32.3	84.8	33.3	139.5	36.5	70.4	118.0	150.7	79.8	87.4
Salvianolic acid	42.9	23.7	9.1	18.6	11.0	27.8	8.0	19.3	28.7	27.5	29.3	90.0
Apigenin	56.27	9.3	10.8	38.3	9.7	10.4	10.3	9.3	10.3	9.1	9.3	12.2
Naringenin	58.13	1.7	3.3	4.3	1.2	6.7	3.5	3.1	4.7	2.9	1.6	0.6
Kaempferol	60.14	0.9	1.3	1.7	0.9	1.5	0.7	nd^b^	3.1	0.9	0.7	1.1

The values are expressed in mg/100 g of sample dry weight.

^a^The data were sorted based on the retention time (RT) of components.

^b^(nd): Not detected.

Among the flavonoids, apigenin was the most abundant one followed by naringenin and epicatechin (Table 1). Apigenin content varied from 9.1 mg/100 g DW in *T. serpyllum* to 38.3 mg/100 g DW in *T. fallax.* Similar range has also been reported in *T. pannonicus*^6^. Several therapeutic properties have been reported for this valuable flavonoid, including cardiovascular, antitumor and bone metabolism effects^39^. On the other hand, rutin has been found as the most abundant flavonoid in *T. glabrescens, T.pannonicus, T. praecox, T. pulegioides, and T. serpyllum*^30^, and high amount of quercetin has also been reported in *T. capitatus*^40^.

Flavonoids are considered as the major group of polyphenolic components with different health benefits. Flavonoids activities are mostly related to their hydroxyl groups as well as the presence of double bounds and their methylation and glycosylation^1^. Flavonoids with an ortho-dihydroxy pattern in the B-ring, such as luteolin and quercetin, are more potent than mono-hydroxylated ones such as apigenin^1^. In the present study, the combination of luteolin-7-glucoside was observed as a derivative. The previous studies showed that activities of glycosylated flavonoids are less than those of aglycons^1^.

### Total phenols and total flavonoid content

*Thymus transcaspicus* possessed the highest phenolic content (37.62 mg gallic acid (GAE) g^−1^ DW), while *T. serpyllum* displayed the lowest amount (22.14 mg GAE g^−1^ DW) (Table 2). Similar range for *T. pulegioides* was obtained using gallic acid as standard for phenolics^13^. However, lower amount (8.1 mg gallic acid equivalents (GAE) g^−1^ DW) was also reported by Roby et al*.*^8^ in *T. vulgaris*. The use of different standards and extraction conditions can highly affect the amount of phenolic content and the extraction yield in different studies^3,31^.

**Table 2:** Total phenolic and flavonoid content of studied *Thymus* species.

Species	TPC^a^ (mg GAE^b^/g DW)	TFC^c^ (mg QE^d^/g DW)
*T. carmanicus*	28.31 ± 0.09	3.81 ± 0.01
*T. daenensis*	26.12 ± 0.06	3.97 ± 0.01
*T. fallax*	31.22 ± 0.05	7.56 ± 0.02
*T. fedtschenkoi*	27.23 ± 0.09	4.34 ± 0.03
*T. kotschyanus*	30.42 ± 0.08	2.97 ± 0.02
*T. migricus*	24.81 ± 0.06	3.91 ± 0.02
*T. pubescens*	30.12 ± 0.07	4.62 ± 0.01
*T. serpyllum*	22.14 ± 0.06	4.36 ± 0.02
*T. transcaspicus*	37.62 ± 0.09	4.75 ± 0.01
*T. trautvetteri*	34.34 ± 0.08	1.77 ± 0.01
*T. vulgaris*	35.73 ± 0.05	8.70 ± 0.01

Means with different letter are statistically significant at 5% level probability.

^a^Total phenolic content.

^b^Gallic acid equivalents.

^c^Total flavonoid content.

^d^Quercetin equivalents.

High variation was found in respect to total flavonoid content (TFC) of 11 studied *Thymus* species. The TFC ranged from 1.77 to 8.72 mg quercetin equivalents (QE) g^−1^ DW. Therefore, the highest and the lowest amounts were obtained in *T. vulgaris* (8.72 mg QE g^−1^ DW) and *T. trautvetteri* (1.77 mg QE g^−1^ DW) species, respectively. Tohidi et al.^45^ also reported similar range for TFC in *T. kotschyanus* (2.11 mg QE g^−1^ DW to 8.14 mg QE g^−1^ DW). Different plant species might have different mechanisms to distribute flavonoids among their subcellular compartments such as accumulation of soluble carbohydrates and the balance between carbohydrate sources and sinks^41^.

### DPPH scavenging test

DPPH is one of the most relevant antioxidant activity tests^42^. The IC_50_ values were found to be in range from 273.8 to 679.3 μg/ml. *T. vulgaris* extract displayed the highest antioxidant activity (the lowest IC_50_ value) (273.8 μg/ml) compared with other species and BHT as synthetic antioxidant, while *T. pubescens* revealed the weakest activity (679.3 μg/ml). The IC_50_ values in studied extracts recorded from 273.8 to 679.3 μg/ml for *T. vulgaris* and *T. pubescens*, respectively (Table 3). IC_50_ differences among extracts could be attributed to various polyphenolic compounds of studied extracts.

**Table 3:** Antioxidant activity of 11 *Thymus* species based on DPPH and β-carotene-linoleic acid model systems.

Species	DPPH scavenging (IC50)	Inhibition of β-carotene bleaching (%)			
		50 ppm	100 ppm	300 pmm	500 ppm
*T. carmanicus*	392.3 ± 0.1	4.14 ± 0.03	11.45 ± 0.09	16.54 ± 0.05	22.28 ± 0.09
*T. daenensis*	371.4 ± 0.1	4.43 ± 0.02	10.88 ± 0.06	16.36 ± 0.04	21.81 ± 0.07
*T. fallax*	369.8 ± 0.1	4.56 ± 0.01	11.81 ± 0.05	17.44 ± 0.06	22.78 ± 0.05
*T. fedtschenkoi*	351.4 ± 0.1	4.67 ± 0.02	13.31 ± 0.04	18.54 ± 0.05	24.49 ± 0.01
*T. kotschyanus*	355.2 ± 0.1	5.66 ± 0.01	13.26 ± 0.06	18.51 ± 0.08	34.48 ± 0.06
*T. migricus*	411.7 ± 0.1	4.39 ± 0.03	7.74 ± 0.08	11.60 ± 0.06	19.03 ± 0.04
*T. pubescens*	679.3 ± 0.1	1.23 ± 0.02	1.59 ± 0.05	2.15 ± 0.07	5.50 ± 0.04
*T. serpyllum*	548.9 ± 0.2	1.99 ± 0.03	6.27 ± 0.09	12.24 ± 0.04	27.42 ± 0.06
*T. transcaspicus*	473.1 ± 0.1	2.85 ± 0.03	7.61 ± 0.07	10.91 ± 0.09	18.25 ± 0.08
*T. trautvetteri*	373.8 ± 0.1	4.55 ± 0.02	11.23 ± 0.05	16.38 ± 0.07	22.20 ± 0.06
*T. vulgaris*	273.8 ± 0.1	7.07 ± 0.04	18.49 ± 0.05	23.68 ± 0.06	43.29 ± 0.07
BHT	172.4	8.21	22.6	30.98	51.34

Means with different letter are statistically significant at 5% level probability.

### Inhibition of β-carotene bleaching

The antioxidant capacity of *Thymus* species based on β-carotene/linoleic acid model system is presented in Table 3. Oxidation of the linoleic acid was inhibited by the extract of *Thymus* species at the concentrations of 500, 300, 100 and 50 ppm (P < 0.05). In this assay, *T. vulgaris and T. kotschyanus* demonstrated higher activities to prevent fatty acid oxidation than other species. The presence of unsaturated fatty acids and their oxidation process is of great importance in food products and diets^43^. Therefore, it is crucial to have a test based on unsaturated fatty acid oxidation in the assessment of antioxidant activity. The mechanism of this analysis can be explained by the phenomenon of “polar paradox”, in which polar antioxidants can be found in the aqueous phase of the emulsion that are more diluted in the lipid phase than aqueous phase and are thus less effective in protecting the linoleic acid^44^.

### Cluster and PCA analyses of polyphenolic compounds

**Figure 2:**
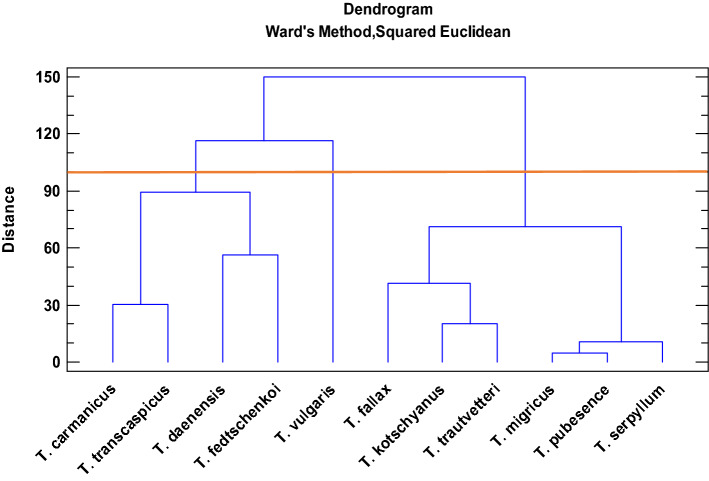
Cluster analysis of studied *Thymus* species according to major polyphenolic profiles.

Cluster analysis was performed to distinguish possible groups among the species using Ward Method (Fig. 2). The hierarchical cluster analysis (HCA) allows subdivision of 11 species into three major groups. Although rosmarinic acid is the most abundant component in most of the species, the cluster analysis did not show a trend for this compound. According to cluster analysis, the first group (*T. carmanicus*, *T. transcaspicus*, *T. fedtschenkoi*, and *T. daenensis*) included medium to high caffeic acid (13.1–27.4 mg/100 g DW) and epicatechin (1.7–1.9 mg/100 g DW). Group 2 (*T. vulgaris*) consisted of high salvianolic acid (90 mg/100 g DW) and low naringenin (0.6 mg/100 g DW), and group 3 (*T. serpyllum*, *T. migricus*, *T. trautvetteri*, *T. fallax*, and *T. kotschyanus*) had high p-coumaric acid (6.3–9 mg/100 g DW) and medium cinnamic acid (4.3–10.7 mg/100 g DW) (Fig. 2). In the present research, groups 1, 2 and 3 were consisted of four, one and six species, respectively (Fig. 2). *T. vulgaris* was grouped in a separate cluster. Therefore, it might be suggested that this species has a different profile in respect to polyphenolic compounds in comparison to other species. In a similar study, Tohidi et al*.*^45^, compared the essential oil composition of *Thymus* species and the cluster analysis for essential oil components, also displayed the similar trend for this species.

**Figure 3:**
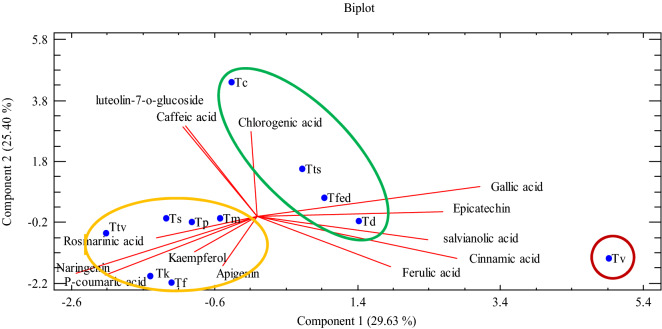
Classification of studied *Thymus* species according to major polyphenolic profiles.

According to PCA, the first three principal components (PC1, PC2, and PC3) explained most of the variation (69.65%). The first PC (PC1) explained 29.63% of total variation and possessed high positive correlation with gallic acid (0.44) and cinnamic acid (0.39) contents, and high negative correlation with naringenin (− 0.36) and p-coumaric acid (− 0.30) contents. PC2 showed 25.40% of total variance and had positive correlation with luteolin-7-o-glucoside (0.44) and caffeic acid (0.44), and high negative correlation with p-coumaric acid (− 0.29) and naringenin (− 0.28) (Fig. 3).

PCA analysis of classified the species into three groups. Group 1 consisted of high rosmarinic acid, naringenin, and p-coumaric acid, while group 2 included species with high contents of chlorogenic acid. *T. vulgaris* was separated from other species in group 3 and was consisted of different components such as salvianolic acid, ferulic acid, cinnamic acid, gallic acid, and epicatechine (Fig. 3).

### Correlations of the compounds

The highest correlation coefficient was obtained between salvianolic acid and ferulic acid (0.79) as well as naringenin and p-coumaric acid (0.76) followed by cinnamic acid and gallic acid (0.69) (Table 4). The highest negative correlation was between naringenin and gallic acid (− 0.74) followed by p-coumaric acid and chlorogenic acid (− 0.52). Most of the compounds showed negative correlation with chlorogenic acid (Table 4).

**Table 4:** Correlation coefficients between phenolic compounds using HPLC on studied *Thymus* species.

Standards													
Galliccacid	1												
Epicatechin	0.470^ns^	1											
Chlorogenic acid	0.092^ns^	0.129^ns^	1										
Caffeic acid	0.023^ns^	− 0.127^ns^	0.635*	1									
Luteolin-7-o-glucoside	0.002^ns^	0.222^ns^	− 0.170^ns^	0.018^ns^	1								
P-coumaric acid	− 0.530^ns^	− 0.471^ns^	− 0.521^ns^	− 0.086^ns^	0.018^ns^	1							
Ferulic acid	0.336^ns^	0.402^ns^	− 0.228^ns^	− 0.239^ns^	− 0.399^ns^	0.119^ns^	1						
Cinnamic acid	0.696*	0.505^ns^	− 0.210^ns^	− 0.480^ns^	− 0.107^ns^	− 0.109ns	0.606*	1					
Rosmarinic acid	− 0.398^ns^	− 0.387^ns^	− 0.066^ns^	0.161^ns^	− 0.280^ns^	0.244^ns^	0.260^ns^	− 0.312^ns^	1				
Salvianolic acid	0.484^ns^	0.398^ns^	− 0.117^ns^	− 0.172^ns^	− 0.294^ns^	− 0.339^ns^	0.798**	0.457^ns^	0.336^ns^	1			
Apigenin	− 0.333^ns^	0.101^ns^	− 0.248^ns^	− 0.242^ns^	− 0.202^ns^	0.231^ns^	0.042^ns^	0.074^ns^	0.004^ns^	0.051^ns^	1		
Naringenin	− 0.746 **	− 0.386^ns^	− 0.218^ns^	− 0.141^ns^	− 0.121^ns^	0.763**	0.058^ns^	− 0.267^ns^	0.415^ns^	− 0.364^ns^	0.225^ns^	1	
Kaempherol	− 0.287^ns^	− 0.123^ns^	0.147^ns^	− 0.116^ns^	0.031^ns^	0.163^ns^	0.118^ns^	0.049^ns^	0.338^ns^	0.094^ns^	0.249^ns^	0.454^ns^	1

*Correlation is significant at the 0.05 level.

**Correlation is significant at the 0.01 level.

ns. Not significant.

Several researches have highlighted the role of phenolic compounds in reducing the risk of many diseases, including neurodegenerative disorders, heart disease and arthritis^46^. However, different kinds of polyphenolic components were determined in studied *Thymus* species in the present analysis. Previous researches have also demonstrated the synergistic or antagonistic effects of flavonoids and phenolic acids with different antixidative activities^31^. Since the mixtures of different polyphenolic compounds are present, as in extracts, the balance between flavonoids and phenolic acids in each species can highly affect its final antioxidant activity. Furthermore, each flavonoid’s concentration can highly affect its final cellular activity^47^. Finally, these modifications and interactions of applied extracts can lead to reduce or increase the final antioxidant activity of food products.

Among studied species, *T. vulgaris* displayed the highest antioxidant activity. This species possessed high amounts of salvianolic acid, ferulic acid, epicatechin and gallic acid. Previous reports emphasized the role of polyphenolic compounds to improve the antioxidative activity of the extracts^48^. In this species, the presence of salvianolic acid and epicatechin might lead to an increased antioxidant activity. High antioxidant activities of flavonoids are reported to attribute to the structure of their rings. Flavonoids possessed three rings, viz A, B and C in which their activity is mostly attributed to the ring B. Furthermore, the presence of hydroxyl group, glycosylation, methylation and the position of double bounds can also affect their activity^1^. In the present study, major flavonoids were epicatechin, apigenin and narenginin (Table 1). Epicatechin belongs to flavanols with two hydroxyl groups in the B-ring, while apigenin has one hydroxyl group. The previous reports illustrated that glycosylated flavonoids, such as luteolin-7-O-glucoside is less potent to scavenge free radicals than aglycons^1^. The highest amount of luteolin-7-O-glucoside was found in *T. fedtschenkoi* (Table 1).

Previous reports have also displayed different antioxidant capacities for phenolic acids and flavonoids. Accordingly, phenolic acids have been introduced as more potent radical scavengers in comparison with flavonoids^41^. Moreover, each model system can be practically different in respect to antioxidant activity in the final product in food industry. For instance, β-carotene-linoleic acid model systems have mostly been used in lipid phase, while DPPH method has been used in non-lipid phases^49^.

### SRAP amplification and levels of polymorphism

The present research evaluated the inter- and intragenic diversity of 11 *Thymus* species for the first time. The primer polymorphisms’ percentage was between 88.88 and 100, representing high diversity among and within *Thymus* species. The mean polymorphic band percentage for the study was 93.83%. The total number of amplified bands per primer ranged from 10 to 19 and averaged 14.57 bands per primer. Amplified products varied from 100 to 800 bp. Me5-em4 marker with 17 bands showed the highest polymorphic band, and Me4-em3 marker with 10 bands revealed the lowest polymorphism. The average polymorphic information content (PIC) value for the amplification products was 0.34. This value was in the range of 0.47 > PIC > 0.23 (Table 5) and the primers displayed a moderate polymorphism according to the previous literature^23^.

**Table 5:** Polymorphism number, annealing temperature and PIC of studied *Thymus* species.

No	Primer	Sequence (5′ → 3′)	Annealing temp^a^	primer combinations	No. total bands	No.PB^b^	PPB^c^ (%)	PIC
1	Me1	5′-TGAGTCCAAACCGGATA-3′	50	Me1 + em4	15	14	93.33	0.38
2	Me2	5′-TGAGTCCAAACCGGAGC-3′	50	Me2 + em1	18	16	88.88	0.32
3	Me3	5′-TGAGTCCAAACCGGAAT-3′	50	Me2 + em3	13	13	100	0.47
4	Me4	5′-TGAGTCCAAACCGGACC-3′	50	Me3 + em1	13	12	92.30	0.33
5	Me5	5′-TGAGTCCAAACCGGAAG-3′	50	Me4 + em3	10	10	100	0.23
6	Em1	5′-GACTGCGTACGAATTAAT-3′	50	Me5 + em3	14	13	92.85	0.28
7	Em3	5′-GACTGCGTACGAATTGAC-3′	50	Me5 + em4	19	17	89.47	0.38
8	Em4	5′-GACTGCGTACGAATTTGA-3′	50					
Total					102	95	–	–
Average					14.57	13.57	93.83	0.34

^a^Degrees celsius (°C).

^b^Number of polymorphic band.

^c^Percentage of polymorphic band.

### Cluster and PCA molecular analyses

**Figure 4:**
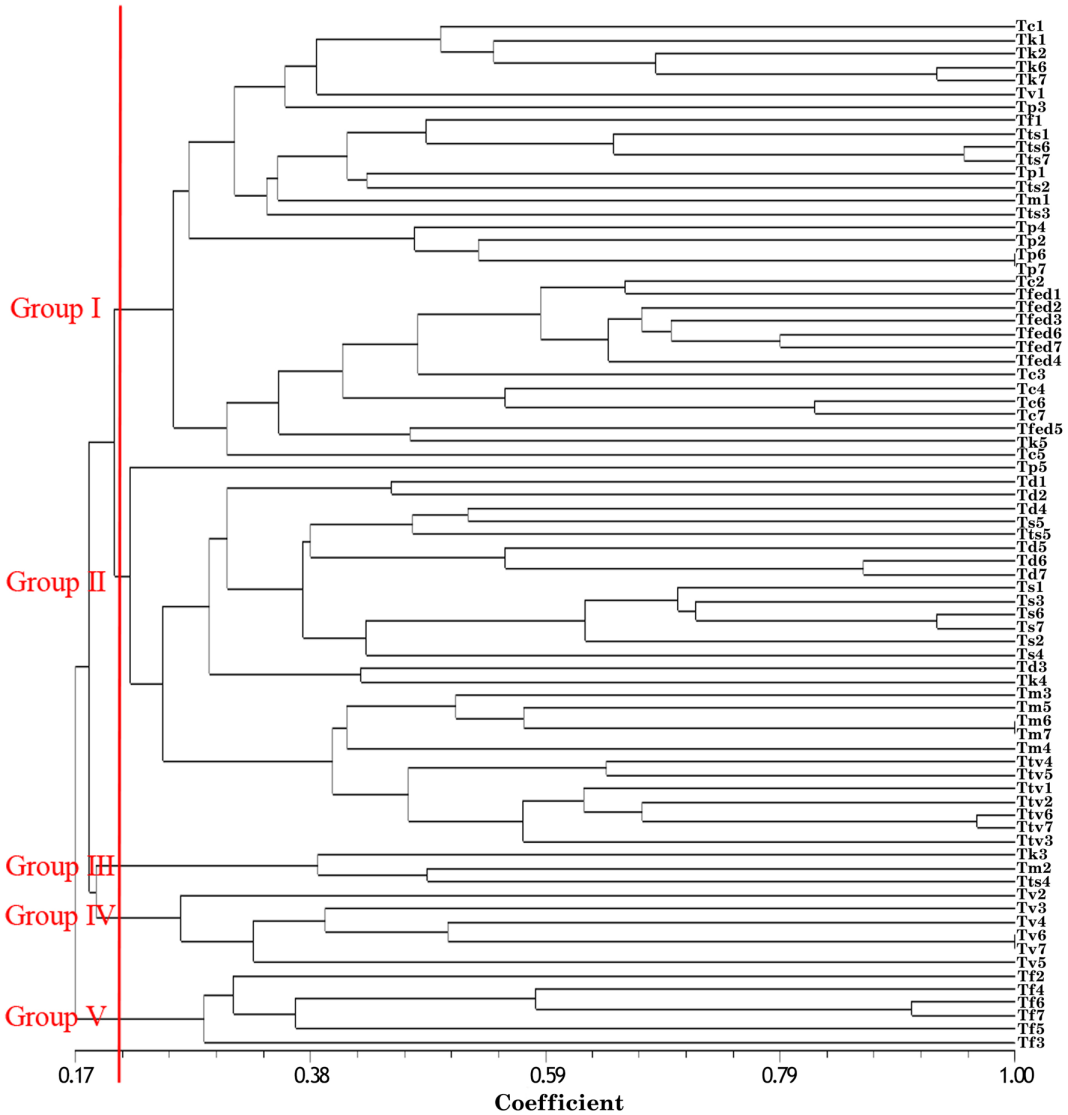
Dendrogram of 77 studied *Thymus* genotypes based on SRAP markers according to the Unweighted Pair Group Mean Algorithm (UPGMA) with the Jaccard similarity index.

The data were also used for cluster analysis. A high cophenetic correlation coefficient of 0.81 between the Jaccard (J) similarity and the cophenetic matrix was obtained accordingly, indicating a good compatibility between the dendrogram and the similarity matrices. Therefore, the Jaccard Method (Fig. 4) determined the dendrogram of genetic relationships among species. This cluster grouped *Thymus* species into five main clusters. The first main cluster (Group I) contained four species, including *T. carmanicus*, *T. transcaspicus*, *T. pubescens*, and *T. fedtschenkoi* (Fig. 4). The second main cluster consisted of *T. daenensis*, *T. serpyllum*, *T. migricus*, *T. trautvetteri* (Group II). The third cluster (Group III) included different species (Fig. 4). The fourth main cluster (Group IV) consisted of *T. vulgaris* accessions (Fig. 4). The last main cluster (Group V) included *T. fallax* (Fig. 4), though the classification did not distinctly separate the species. For instance, *T. carmanicus*, *T. fedtschenkoi*, *T. daenensis*, and *T. serpyllum* were classified in one group. This kind of discrepancy in clusters might be interpreted by ploidy level, cross pollination, high gene flow and natural hybridization^50^.

**Figure 5:**
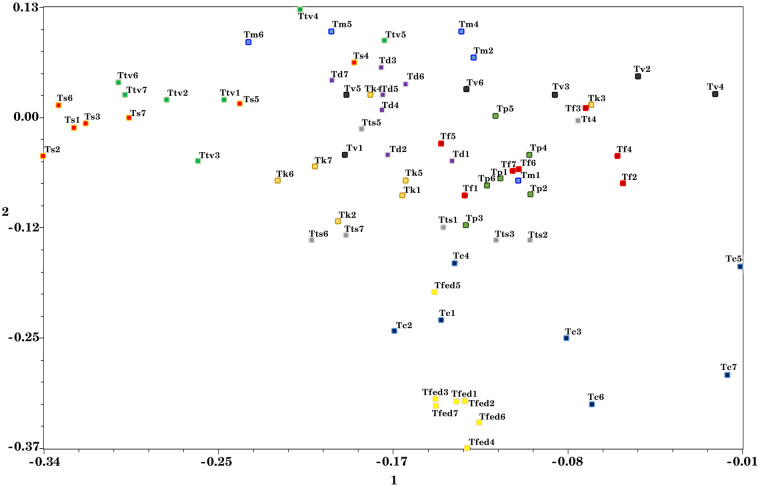
Plot of *Thymus* samples by principal coordinate analysis using the Jaccard’s similarity coefficients. Footnote: *T. migricus*; *T. fallax*; *T. carmanicus*; *T. kotschyanus*; *T. pubescens*; *T. daenensis*, *T. trautvetteri*; *T. transcaspicus*; *T. fedtschenkoi*; *T. vulgaris*; *T. serpyllum*.

The PCoA results of the SRAP were also performed to confirm the cluster analysis. The PcoA results were largely in line with those obtained by cluster. They revealed that the first three principal coordinates (PCo) accounted for 37.8% of the total variation (Fig. 5) which suggests the relatively high distribution of markers throughout the genome.

### Analysis of molecular variance (AMOVA) and admixture of species

AMOVA was also used to estimate the percentage of intra and inter species genetic variations (Table 6). AMOVA results demonstrated significant variations among the species (P < 0.001), so that 63.14% of total genetic variation occurs in SRAP analysis within the species. In addition, the total genetic variation among the species was equal to 36.86% in the SRAP analysis. The analysis also revealed a relatively acceptable differentiation in allele frequencies (Fst = 0.36) and a relatively high gene flow (Nm = 0.63) among the studied species. Furthermore, most of genetic variation was within the species (Table 6). Table 6 illustrates the species level of genetic diversity indices of genetic flow (Nm), and diversity among species (gene differentiation factor) (Gst).

**Table 6:** Analysis of molecular variance (AMOVA) among and within *Thymus* species using SRAP markers.

Source of variation	*df*^a^	Mean of squares	Percentage of variation	*P* value	Fst	Nm^b^	Gst^c^
Among species	10	22.24	36.86	< 0.001			
Within species	66	4.37	63.14				
Total	76	6.72			0.36	0.63	0.44

^a^Degrees of freedom.

^b^Gene flow. Nm = estimate of gene flow from Gst or Gcs. E.g., Nm = 0.5(1—Gst)/Gst; McDermott and McDonald^58^.

^c^Diversity among species.

Moreover, the high variation within the studied *Thymus* species could be attributed to different factors, including the recombination of genes and gene mutation^51^. In addition, some environmental factors can affect the variability of the species. For example, the genetic exchange among the species can be a result of the mobility degree of the pollinator^16^.

**Figure 6:**
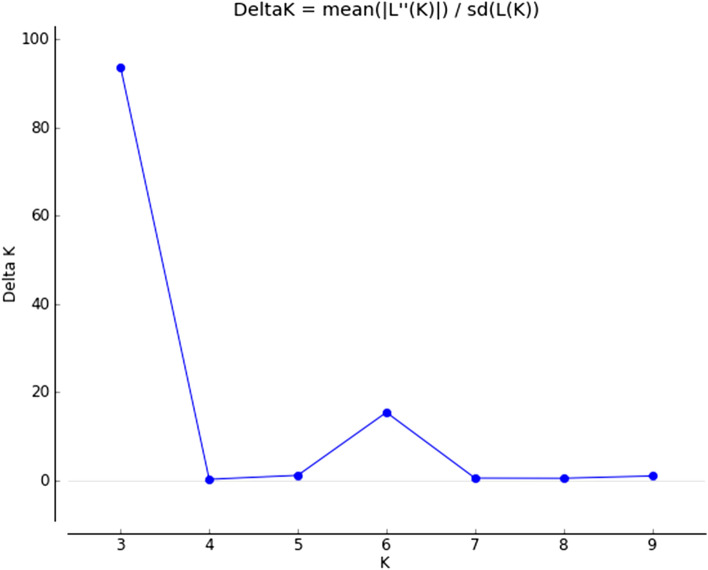
Estimation of ΔK value obtained with STRUCTURE analysis.

**Figure 7:**
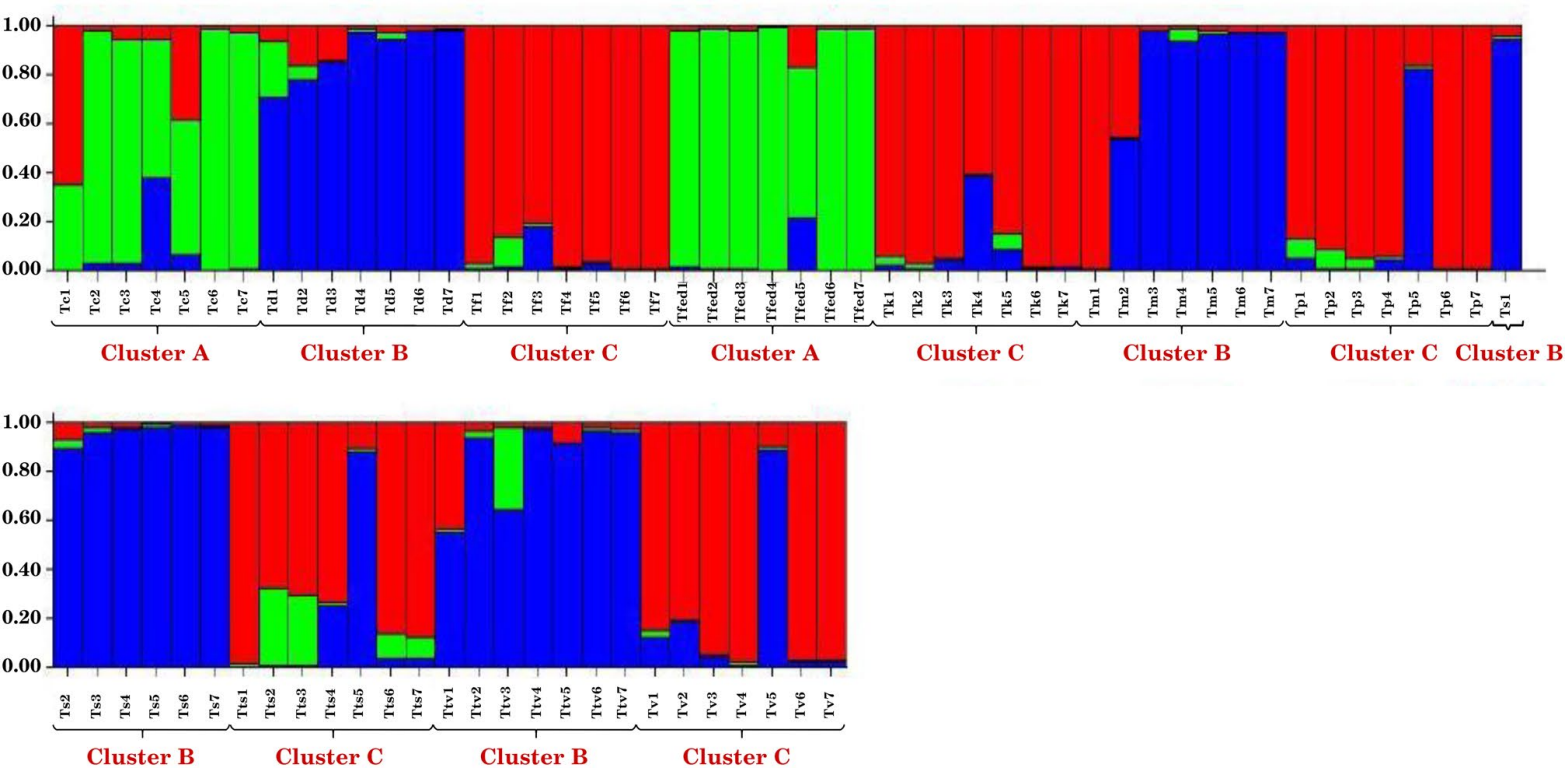
Genetic structure of 77 *Thymus* genotypes as inferred by STRUCTURE software with eight SRAP markers data set. Single vertical line represents an individual accessions and different colors represent genetic stocks/gene pools. Segments of each vertical line show extent of admixture in an individual (for interpretation of the references to colour in the text, the reader is referred to the web version of this article).

STRUCTURE analysis was also carried out for a better interpretation of admixture in some species obtained through cluster and PCA. The value of K was calculated by posterior probability of the data for a given K, Pr (X|K)^52^. Accordingly, the highest K value was estimated as K = 3 (Fig. 6). Therefore, analysis was done based on three colors viz. green (A), blue (B) and red (C) for studied *Thymus* species as shown in Fig. 7 Cluster A in Fig. 7 contained the species including *T. carmanicus* and *T. fedtschenkoi*. Cluster B included species such as *T. daenensis*, *T. migricus*, *T. serpyllum*, and *T. trautvetteri*. The remaining species containing *T. fallax*, *T. kotschyanus*, *T. pubescens*, *T. transcaspicus*, and *T. vulgaris* were classified in cluster C. Low to moderate admixtures were observed in the analysis. However, some species revealed higher admixture in comparison with others. For instance, *T. carmanicus* and *T. transcaspicus* showed high admixture in this research. One probable reason for this admixture might be a result of natural hybridization in some *Thymus* species due to the lack of incompatibility mechanism and other self-pollination strategies.

Comparing molecular and phytochemical clusters revealed some similarities in two dendrograms. For instance, *T. vulgaris*, separated from other species according to major polyphenolic profiles and molecular analysis, as well as *T. transcaspicus*, *T. carmanicus*, and *T. fedtschenkoi*, were clustered in the same groups. Consequently, this similarity would lead to another hypothesis that the evolution of some phenolic components in *Thymus* species can be caused by the effect of genetic and epigenetic factors simultaneously.

Overall, based on the objectives of the research, phenolic and flavonoid compounds of many *Thymus* species were determined and compared with SRAP molecular classifications for the first time. Furthermore, in the present research, the classifications based on molecular and phytochemical revealed similar trend in some species.

## Conclusion

A high variation was found among *Thymus* species in respect to both molecular and phytochemical aspects consequently. High inter and intra genetic variation was obtained in studied species using SRAP markers. Among the species, *T. vulgaris* had the highest amount of salvianolic acid and antioxidant activity. Moreover*,* the phenolic compounds and flavonoids in studied species can introduce elite species for pharmaceutical and food purposes. The results of antioxidants study demonstrated that *T. vulgaris*, *T. fedtschenkoi*, and *T. kotschyanus* had the strongest antioxidant activity among the 11 species, while *T. transcaspicus*, *T. vulgaris*, and *T. trautvetteri* were found to be rich in total phenolic content. Overall, *T. vulgaris* and *T. fallax* may be recommended as good sources of phenolic compounds and flavonoid content with a high antioxidant activity. Finally, based on molecular and phytochemical analyses, similar trends were observed in *T. transcaspicus*, *T. carmanicus*, and *T. fedtschenkoi* species.

## Materials and method

### Plant material

The seeds of 11 *Thymus* species (*T. vulgaris*, *T. fallax*, *T. trautvetteri*, *T. transcaspicus*, *T. fedtschenkoi*, *T. daenensis*, *T. carmanicus, T. pubescens, T. serpyllum, T. kotschyanus*, and *T. migricus*) were obtained from gene bank of Research Institute of Forests and Rangeland, Tehran. The species were identified using Flora Iranica^53^ by Dr. Mozzafarian. The accession codes and their collection sites are illustrated in Table 7. The seeds were grown in a randomized block design (RCBD) with three replications.

**Table 7:** Gene bank codes, accession codes and origins of *Thymus* species.

Accession no	Gene bank codes	Accession codes	Species	Origin
1	18316	Tc	*T. carmanicus*	Zarand, Kerman, Iran
2	10126	Td	*T. daenensis*	Fereydonshahr, Isfahan, Iran
3	33916	Tf	*T. fallax*	Shahedieh, Yazd, Iran
4	18063	Tfed	*T. fedtschenkoi*	Naqadeh, Azarbaijan Gharbi, Iran
5	17090	Tk	*T. kotschyanus*	Qazvin, Qazvin, Iran
6	33918	Tm	*T. migricus*	Shahedieh, Yazd, Iran
7	39341	Tp	*T. pubescens*	Shahedieh, Yazd, Iran
8	33920	Ts	*T. serpyllum*	Shahedieh, Yazd, Iran
9	3507	Tts	*T. transcaspicus*	Khorasan Razavi, Hezar Masjed, Iran
10	33921	Ttv	*T. trautvetteri*	Shahedieh, Yazd, Iran
11	14287	Tv	*T. vulgaris*	London, England, United Kingdom

### Preparation of the methanolic extract

The aerial parts *Thymus* species were harvested at flowering stage. The harvested parts were shade dried at 25 °C, and the Plant materials were ground and prepared for extraction. Methanol was extracted using 50 g of the dried sample. The extract was shaken using 500 mL of methanol with 150 rpm at 25 ºC for 72 h. Then, a three-layer cheesecloth to remove any solid contamination filtered the extract. Finally, the extracts were evaporated at room temperature (25˚C) and dried in desiccators under vacuum to a constant weight.

### High performance liquid chromatography (HPLC)

The studied *Thymus* extracts were analyzed by the HPLC system (model Agilent 1090). The HPLC elution method has been used previously by Gharibi et al.^1^. Solutions of pure known compounds available to the study were used for chromatography as external standards. All standards (gallic acid, epicatechin, chlorogenic acid, caffeic acid, p-coumaric acid, ferulic acid, cinnamic acid, rosmarinic acid, salvianolic acid, apigenin, naringenin, and kaempferol) were dissolved in HPLC grade methanol before injection to the analytical HPLC system. A 0.22 μm nylon acro-disk filter and 20 μL of the extract were used for injection. The stationary phase had a 250 mm × 4.6 mm (5 μm) symmetry C18 column (Waters Crop., Milford, MA, USA) (10 mm × 4 mm I.D.), and the mobile phase included solvent A and B with a flow rate of 0.8 mL min^−1^ and the detection was performed between 200 and 400 nm through UV detector. The column was 25 °C. 0.1% of water-formic acid was applied as solvent A, while 0.1% of formic acid in acetonitrile was used as solvent B in the mobile phase. The gradient conditions were also performed as follows: a linear step from 10 to 26% solvent B (v/v) for 40 min, 65% solvent B for 70 min, and finally to 100% solvent B for 75 min. The phenolic compounds were calculated comparing the peak areas and their retention times. Finally, the results were reported as mg/100 g of the sample dry weight.

### Determination of total phenolic and flavonoid contents

Total phenolic content (TPC) was evaluated using Folin-Ciocalteu method. In this procedure, the methanolic extract (0.5 ml) was mixed with tenfold-diluted reagent (2.5 ml) and 7.5% sodium carbonate (2 ml) and then heated at 45 °C for 15 min. Then, absorption was measured using spectrophotometer (Hitachi U-1800) at 765 nm against a blank and the phenolic content was obtained as mg of equivalent tannic acid per gram of dry weight. Total flavonoid content (TFC) of the methanolic extract was determined by Gharibi et al*.*^31^ procedure, using the aluminum chloride colorimetric method. In the present study, sodium hydroxide 4% solutions, sodium nitrate 5%, and aluminum chloride 10% were applied. The extraction yield was evaluated as the ratio of the extract weight and the dry weight of each species^43^.

### DPPH scavenging activity

According to the radical scavenging effect on the DPPH free radicals, the plant leaf extracts’ antioxidant activity, as well as standard antioxidants were assessed (Table 3). Different concentrations of *Thymus* extracts (equivalent to 50, 100, 300 and 500 ppm) were provided in methanol. BHT was used as the synthetic antioxidant (of 0.1 ml sample solution) with five milliliters of 0.1 mM methanolic solution of DPPH that were mixed separately. The mixtures were protected in dark condition for 30 min and the optical density was also evaluated by spectrophotometer (Hitachi U-1800) at 517 nm. The antioxidant activity was calculated based on radical-scavenging activity^41^.

### Antioxidant activity using β-carotene-linoleic acid model system

This study is carried out based on the method explained by Salami et al*.*^43^. For this purpose, 0.5 mg β-carotene is dissolved in 1 ml of chloroform and 200 mg Tween 80, and 25 µl linoleic acid was added to provide the stock solution. The solvent evaporated and 100 ml ddH_2_O (double-distilled water) was added. Afterwards, BHT was applied as synthetic antioxidant in the concentration of (0.5–5 mg per 1 ml) at 50 °C. The absorbance of the sample extract was calculated using spectrophotometer (Hitachi U-1800) at zero time (t = 0) at 490 nm. Absorbance was read at an interval of 25 min (t = 125 min). Antioxidant capacity was stated as the percent of inhibition based on^54^ reports.

### DNA extraction

In the present research, 77 accessions belonging to 11 *Thymus* species (seven individual plants from each species) were used. DNA extraction was carried out using modified CTAB procedure^55^. For the estimation of DNA concentration, spectrophotometer UV visible (Hitachi U-1800), as well as gel electrophoresis were applied.

### SRAP analysis

PCR reaction was performed by eight selected primer combinations in thyme. For this purpose, a reaction of 15 μl was used. 7 μl of Master Mix Red (Ampliqon, Finland) was used in reaction along with 4 mM MgCl_2_, 1× PCR buffer, 15 ng DNA, 10 pM of each primer (Me and Em combinations), and 1.5 μl of ddH_2_O. After selecting the best annealing temperatures, PCR was performed as follows: initial denaturation (94 °C for 1 min) was followed by 35 cycles of 94 °C for 1 min, 50 °C for 1 min, and 72 °C for 1 min. Finally, five minutes at 72° was used as final extension. DNA products were separated in a 1× TBE (100 mMTris–Borate, pH = 8.0 and 2 mM EDTA), 7% acrylamide gel at 220 V for three hours and finally stained by silver nitrate.

### Data analysis

The cluster and PCA were performed by NTSYSpc, Ver. 2.02^56^. Genetic relationships of samples were calculated by the Jaccard’s Similarity Index^57^ through the Simqual routine. The genetic structure parameters were assessed using Pop Gene. ver. 32^58^ (Table 8). Variation among and within species were evaluated using Arlequin ver. 3 software. Admixture of *Thymus* species was assessed using Structure Software based on Evanno’s Method^59^ The K value was computed with the probability Posterior of a K value, Pr (X | K)^52^. Variance analysis was performed using SAS ver.9.2. Statgraphic ver.16.2.04 also was applied for PCA and dendrogram production.

**Table 8:** Summary of genetic variation statistics for SRAP markers used in *Thymus* species.

Species	Sample size	Na^a^	Ne^b^	H^c^	I^d^	NPL^e^	PPL^f^ (%)	He^g^
*T. carmanicus*	7	1.66	1.34	0.21	0.33	68	66.67	0.25
*T. daenensis*	7	1.43	1.20	0.13	0.20	44	43.14	0.15
*T. fallax*	7	1.42	1.17	0.12	0.19	43	42.16	0.14
*T. fedtschenkoi*	7	1.45	1.18	0.12	0.20	46	45.10	0.15
*T. kotschyanus*	7	1.46	1.26	0.16	0.24	47	46.08	0.19
*T. migricus*	7	1.58	1.28	0.18	0.29	60	58.82	0.21
*T. pubescens*	7	1.45	1.23	0.14	0.22	46	45.10	0.17
*T. serpyllum*	7	1.43	1.21	0.14	0.21	44	43.14	0.16
*T. transcaspicus*	7	1.57	1.29	0.18	0.28	59	57.84	0.21
*T. trautvetteri*	7	1.48	1.23	0.15	0.23	49	48.04	0.17
*T. vulgaris*	7	1.53	1.23	0.15	0.24	55	53.92	0.18
Multi population	77	2	1.45	0.28	0.44	–	–	0.28

^a^Observed number of alleles.

^b^Effective number of alleles.

^c^Gene diversity.

^d^Shannon’s information index.

^e^Number of polymorphic loci.

^f^Percentage of polymorphic loci.

^g^Expected heterozygosity.
